# Thiol Modification of Psyllium Husk Mucilage and Evaluation of Its Mucoadhesive Applications

**DOI:** 10.1155/2013/284182

**Published:** 2013-11-20

**Authors:** Meenakshi Bhatia, Munish Ahuja

**Affiliations:** Drug Delivery Research Laboratory, Department of Pharmaceutical Sciences, Guru Jambheshwar University of Science and Technology, Hisar 125 001, India

## Abstract

Thiol functionalization of psyllium was carried out to enhance its mucoadhesive potential. Thiolation of psyllium was achieved by esterification with thioglycolic acid. Thiolation was observed to change the surface morphology of psyllium from fibrous to granular and result in a slight increase in the crystallinity and swelling. Thiolated psyllium was found to contain 3.282 m moles of thiol groups/g of the polymer. Mucoadhesive applications of thiolated psylium were explored by formulating gels using metronidazole as the model drug. On comparative evaluation thiolated psyllium gels showed 3-fold higher mucoadhesive strength than the psyllium gels as determined by modified physical balance using chicken buccal pouch. The results of *in vitro* release study revealed that thiolated psyllium gels provided a prolonged release of metronidazole. Further, the psyllium and thiolated psyllium gels were found to release the drug following first-order kinetics by combination of polymer relaxation and diffusion through the matrix.

## 1. Introduction

Natural polysaccharides and their derivatives are extensively used in pharmaceutical and food industry as biodegradable and biocompatible polymers for a large number of applications such as binding, thickening, emulsifying, and gelling and as controlled release agents. Psyllium is one such polysaccharide which has been used since times immemorial as a dietary supplement to promote the regulation of bowel movement. Psyllium also known as is paghula is comprised of seed husks of *Plantago ovata* Forsk (family Plantaginaceae). The laxative and long acting action of *Plantago* husk has been attributed to the gel forming fraction of the husk. Psyllium mucilage obtained from psyllium husk is white fibrous hydrophilic material that forms clear colorless mucilaginous gel by absorbing water.

The polysaccharides extracted from the husk of *Plantago ovata* have been chemically characterized to contain a high proportion of hemicellulose which is the alkali soluble fraction of the husk. It consists of highly branched acidic arabinoxylan comprising of xylan backbone chain with xylose and arabinose forming the side chains [1].

In recent years, chemical modification of natural polysaccharides to improve their properties has received considerable interest. Thiol modification of natural polysaccharides such as chitosan, alginate, pectin, and tamarind seed xyloglucan has been successfully done to improve their functional properties [2–5]. Psyllium has shown potential to be exploited as safe and effective drug carrier in pharmaceutical industry. Psyllium and its chemically modified derivatives have been explored for modification of water absorbing, gelling, and release properties [6]. In pharmaceutical applications psyllium and its modifications have earlier been explored for preparing sustained release matrix and colon specific drug delivery [7, 8]. However, some of the chemical modifications of psyllium have been discussed, but the thiolation of psyllium is not yet explored. The paper describes thiol modification of psyllium and its characterization and evaluation for mucoadhesive applications.

Thiolated psyllium was characterized by Fourier transform infrared spectroscopy (FT-IR), thermogravimetric analysis (TGA), X-ray diffraction analysis (XRD), and scanning electron microscopy (SEM). The numbers of thiol group substituents/gram of polymer were determined by Ellman's method. Thiolated psyllium was further explored for mucoadhesive applications by formulating gels, employing metronidazole as the model drug. Further, the gels were characterized mechanically by texture analysis. Mucoadhesive strength of metronidazole loaded psyllium and thiolated psyllium gels were comparatively evaluated using freshly excised chicken buccal pouch by modified physical balance method. 

## 2. Materials and Methods

### 2.1. Chemicals and Materials

 Psyllium seed husk (Sidpur Sat Isabgol factory, Gujarat, India) was purchased from local market. Metronidazole was obtained as gift sample from Ranbaxy Laboratories (Gurgaon, India). Thioglycolic acid, hydrochloric acid, methanol, and sodium hydroxide were procured from SD Fine-Chem Ltd. (Mumbai, India) and used as received. Freshly excised chicken buccal pouch was obtained from the local butcher shop (Hisar, India). Commercial formulation of metronidazole gel (Metrogyl, Lekar Pharmaceuticals, Ankleshwar, India) was purchased from the local pharmacy store.

### 2.2. Synthesis of ** **Thiolated Psyllium (TPSY)

TPSY was synthesized by the esterification of psyllium (PSY) with thioglycolic acid in the presence of hydrochloric acid. The reaction was carried out with 2 moles of thioglycolic acid for every 1 mole of hydroxyl group in PSY [9]. Psyllium husk was soaked in water (1 : 50) for overnight and the pH of the mixture was adjusted to 12 with sodium hydroxide (2.5% w/v) solution. The insoluble fraction was removed by passing it through muslin cloth. The reaction between husk free PSY and thioglycolic acid was carried out in the presence of catalytic amount of hydrochloric acid at 70°C for 90 min. The reaction mixture was cooled and precipitated by adding methanol followed by washing with methanol to remove unreacted thioglycolic acid. TPSY so obtained was frozen at −80°C for 4 h followed by lyophilization in a laboratory model freeze drier (Alpha 2-4 LD Plus, Martin Christ, Germany) at −90°C and 0.0010 mbar pressure for 24 h.

### 2.3. Characterization

*Fourier Transform Infrared Spectroscopy (FTIR).* PSY and TPSY samples were subjected to FT-IR spectroscopy in a Fourier-transform infrared spectrophotometer (Perkin-Elmer, spectrum) in range of 4000 cm^−1^ and 500 cm^−1^ using KBr pellet method. 

*Thermal Analysis.* Thermogravimetric analysis (TGA) and differential scanning calorimetery (DSC) of PSY and TPSY were recorded using a simultaneous thermal analyser (SDT, Q 600, TA instruments, USA) in a temperature range of 25 and 600°C under constant nitrogen purge of 100 mL/min at a heating rate of 10°C per min.

*Powder X-Ray Diffraction Analysis (PXRD).* The PSY and TPSY powder samples were scanned using an X-ray diffractometer (Miniflex 2, Rigaku, Japan) from 0°to 80° diffraction angle (2*θ*) range under the following measurement conditions: source, nickel filtered Cu-K*α* radiation; voltage 35 kV; current 25 mA; scan speed 0.05 min^−1^; division slit 1.25°; receiving slit 0.3 mm. 

*Scanning Electron Microscopy (SEM). *The shape and surface morphology of PSY and TPSY were investigated using scanning electron microscope (JEOL, JSM-6100). The samples were coated with gold and mounted on a sample holder. The electron micrographs were taken at an accelerating voltage of 10 kV. 

*Determination of  Thiol Substitution. *The degree of thiol group substitution was determined by quantifying the amount of thiol groups in TPSY by Ellman's method [4]. Aqueous dispersions (0.5%, w/v) of TPSY and PSY (as control) were prepared and diluted with phosphate buffer (5M, pH 8.0) to a concentration of 0.15% (w/v). An aliquot of 5 mL of the polymer solution was allowed to react with 5 mL of Ellman's reagent (0.3% w/v) for 2 h at room temperature, followed by measurement of absorbance of the reaction mixture at 450 nm. The numbers of thiol group substituents per gram of TPSY were determined using a calibration curve prepared by reacting standard solutions of thioglycolic acid with Ellman's reagent as previously detailed.

*Swelling Power. *One gm of PSY and TPSY was transferred to a 100 mL glass stoppered cylinder containing 90 mL of water, shaken well for 30 s, and allowed to stand 24 h. The cylinders were shaken gently on three occasions during the 24 h of study. A sufficient quantity of water was added to produce 100 mL and mixed gently for 30 s and was allowed to stand for further 5 h. The volume occupied by the swollen mucilage was measured and swelling power was noted.

### 2.4. Evaluation of TPSY as Mucoadhesive Polymer

#### 2.4.1. Formulation of Gels

TPSY was evaluated for mucoadhesive applications by formulating metronidazole gels. Powders of PSY and TPSY were dispersed at a concentration of 2% (w/v) in aqueous solution of metronidazole (1%, w/v) and allowed to hydrate overnight to form gels.

#### 2.4.2. Physicochemical Characterization of Gels

*Viscosity. *Viscosity of gel formulations was determined by Brookfield viscometer (Brookfield DV-E Viscometer) at various speeds using spindle number 6.

*Mechanical Characterization of Gels. *Structural analysis of PSY and TPSY gels was done to determine their mechanical properties such as hardness, cohesiveness, and adhesiveness. Mechanical characterization was conducted employing a software-controlled penetrometer (TA-XT2, Stable Micro Systems, UK) by measuring the resistance to penetration and withdrawal of the cylindrical aluminium probe (dia 22 mm). The penetrometer was equipped with a 5 Kg load cell. The pretest speed was set up at 5 mm/s, the test speed at 1 mm/s, and the penetration depth at 5 mm, with an acquisition rate of 100 points/s. The study was carried out at room temperature [10]. Hardness was measured as the height of the first positive peak on force time curve while area of first negative peak gives the adhesiveness of the formulation. The cohesiveness is calculated as the ratio of area of second positive peak to first positive peak.

*Mucoadhesive Strength.* Mucoadhesive strength of PSY and TPSY gels was assessed using modified physical balance method. This apparatus was comprised of a tared two-pan physical balance. A glass slide was glued to the bottom of one of the pans while another glass slide was glued to the base of the stage beneath the pan. To tare the balance, another glass slide was glued to the other pan of the balance. A freshly excised chicken buccal pouch was collected within 30 min of slaughter from the local slaughter house and transported to laboratory in cold normal saline. The buccal mucosal membrane was carefully excised and separated from the adhering tissues followed by washing with normal saline. The buccal mucosal membrane was glued to both glass slides. An accurately weighed gel formulation (1 g) was sandwiched between the chicken buccal tissues. The pans of the balance were kept on the resting stage. A preload force of 50 g was applied for 5 min on the pan above the tissue. After removal of the preload force, the balance was lifted by holding the lever and a gradually increasing weight was applied on the other pan of the balance by controlled addition of water till the plates were detached from each other. The weight (g) of the water required for the detachment of the glass plates was recorded and the force of detachment (N) was calculated and taken as an index of the mucoadhesive strength of gels [11].

*Mucoadhesion Retention Time. *Mucoadhesion retention time of PSY and TSPY gels was comparatively evaluated by applying 1 g of the gels containing a dye, chlorophenol red (0.01%, w/v) on a chicken buccal tissue glued to a glass slide. The slide was placed at an angle of 45° and was dripped with Mcllvaine buffer (pH 6.6) at a rate of 30 mL/min. The time required for complete removal of dyed gel from the tissue was recorded by visual examination.

*In Vitro Drug Release Study*. *In vitro* release of metronidazole from various gel formulations was carried out by placing an accurately weighed gel containing equivalent to 10 mg of metronidazole in the dialysis tubing. The dialysis tube was tied to the paddle of USP type II dissolution apparatus (TDL-08L, Electrolab, India) and immersed in 500 mL of dissolution medium (Mcllvaine buffer pH 6.6). The dissolution media was maintained at 37°C with continuous stirring at 100 rpm. A 5 mL sample was withdrawn at various time intervals and the media volume was maintained by adding equal volumes of fresh media [11]. The concentration of metronidazole in the samples was determined spectrophotometrically by measuring absorbance at 318 nm and calculating the concentration employing the calibration curve prepared over the linearity range of 2–40 *μ*g/mL, with the equation of line being Y = 0.0519X + 0.0452 (*R* ^2^ = 0.9913). 

## 3. Results and Discussion

The covalent attachment of thioglycolic acid to psyllium was achieved by ester bond formation between hydroxyl group of arabinoxylan moieties of psyllium and carboxyl group of thioglycolic acid. After being ground in a mortar, product appeared as off-white odorless powder, soluble in water. The average yield of this synthesis amounted to 91.8% of the utilized amount of psyllium. The product was found to contain 3.282 ± 1.051 m moles of thiol groups/gram as determined by Ellman's method.

**Figure 1:**
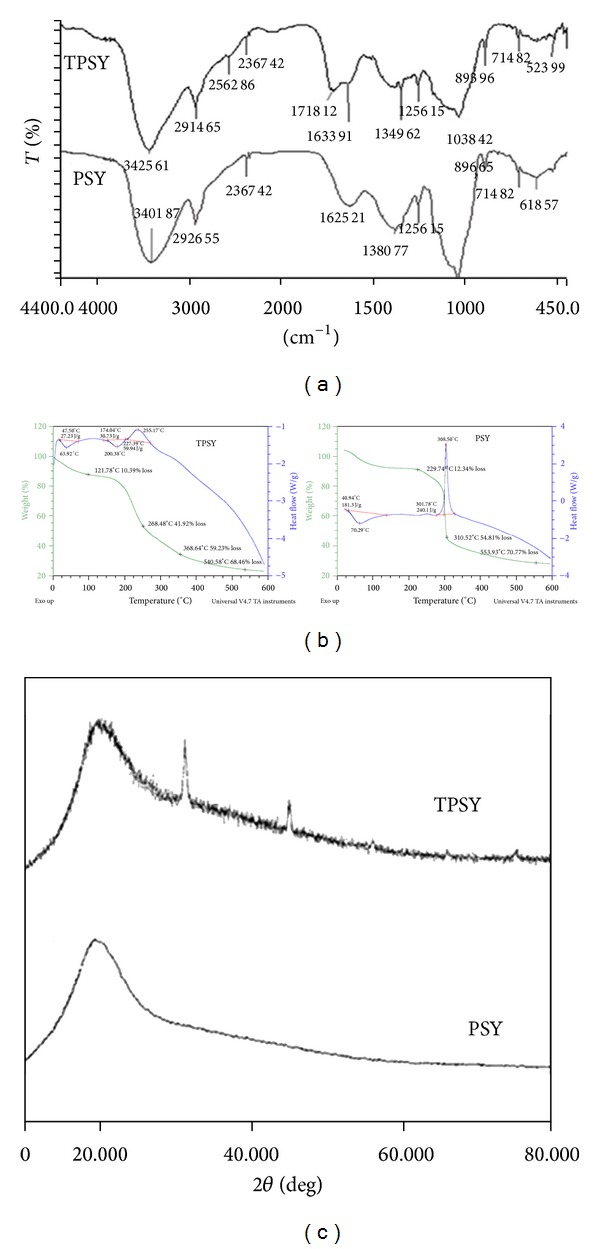
(a) FTIR spectra of PSY and TPSY. (b) Thermal curve of PSY and TPSY. (c) XRD spectra of PSY and TPSY.

Figure 1(a) exhibits the FT-IR spectrum of PSY and TPSY in the frequency region from 4000 to 500 cm^−1^. The spectra of PSY shows a broad absorption band at 3401 cm^−1^ which can be attributed to –OH stretching of alcohols. A peak appearing at 2926 cm^−1^ is due to –CH stretching of alkanes, while the peak at 1050 cm^−1^ was ascribed to C–O–C stretch of ether. The peaks appearing at 896, 714, and 613 cm^−1^ may be due to polymer backbone bendings. The IR spectra of TPSY show the absorption band due to OH stretching at 3425 cm^−1^, while the –SH stretch appears at 2562 cm^−1^ which confirms the thiolation. The C–O–C stretch appears at 1038 cm^−1^ in TPSY, while the peaks at 893, 714, and 523 cm^−1^ are for polymer backbone bendings.

Figure 1(b) shows the TGA and DSC thermograms of PSY and TPSY. The DSC curve of psyllium shows a broad endotherm at 70.29°C with heat of fusion of 181.3 J/g followed by an exotherm at 308.50°C with heat flow of 240.1 J/g. The DSC thermogram of TPSY shows a broad endotherm at 63.92°C and 200.38°C with heat of fusion of 27.23 J/g and 30.73 J/g, respectively. Thus, a decrease in the endothermic transition temperature, disappearance of exotherm, and appearance of one more endotherm in TPSY were obtained on thiolation of PSY. Further the results of thermogravimetric analysis of PSY revealed two major stages of decomposition. The first stage was characterized by the initial decomposition temperature (IDT) of 229°C and final decomposition temperature (FDT) of 310°C. The first stage corresponds to major breakdown of polymer chain which resulted in 54.81% weight loss accompanied by a large exothermic enthalpy change which may be attributed to degradation of PSY with evolution of carbon dioxide and water vapors and so forth. The second stage of decomposition was characterized by degradation temperature of 553°C with 70.7% weight loss. The TGA curve of TPSY also reveals two major stages of decomposition. The first stage is characterized by IDT of 121.78°C and FDT of 268.48°C accompanied by small exothermic enthalpy change. Further 41.92% of weight loss was observed during first decomposition stage. The second decomposition stage of TPSY was characterized by IDT of 368°C and FDT of 540.58°C with weight loss of 68.46%. 

Figure 1(c) displays the X-ray diffraction spectra of PSY and TPSY. X-ray diffractogram of PSY is typical of amorphous materials with no sharp peaks while the diffractgram of TPSY shows two characteristic sharp peaks at 32° and 45° (2*θ*) which indicates slight increase in crystallinity of PSY on thiolation.

**Figure 2:**
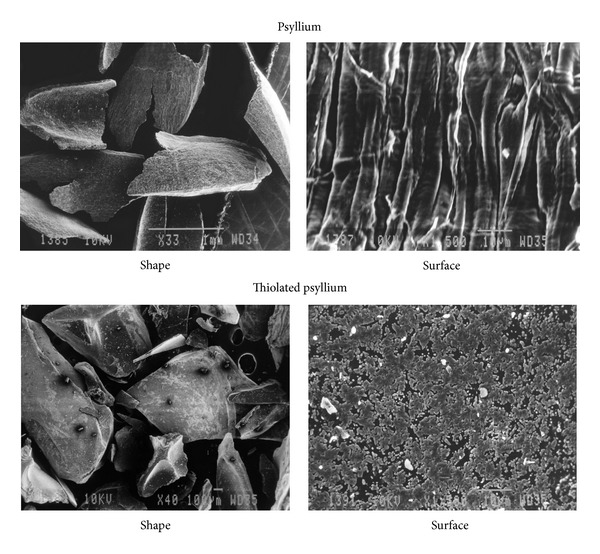
Scanning electron micrographs showing shape and surface of PSY and shape and surface of TPSY.

Figure 2 shows the shape and surface morphology of PSY and TPSY, examined under a scanning electron microscope. The shape of PSY and TPSY particles was found to be polyhedral. A close examination of surface morphology reveals that surface of PSY is fibrous while the surface of TPSY is granular. 

PSY possesses good swelling property. Swelling power studies conducted on PSY and TPSY revealed that 1 g of PSY and TPSY on hydration in water swells to 86.6 and 100 mL, respectively. Thus, thiolation of PSY results in 1.16- fold increase in swelling power. 

**Figure 3:**
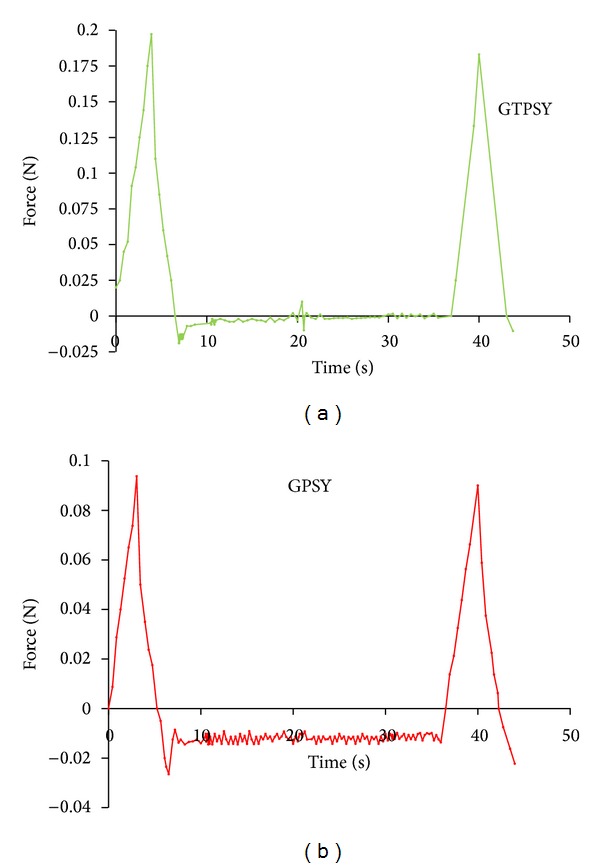
Texture profile analysis of GPSY and GTPSY gels.

The mucoadhesive applications of synthesized TPSY were comparatively evaluated by formulating gels of PSY and TPSY employing metronidazole as the model drug. The gels were prepared by adding PSY or TPSY at concentration of 2% (w/v) in aqueous solution of metronidazole 1% (w/v) under constant stirring and allowing them to hydrate overnight. The metronidazole loaded PSY gel (GPSY) and TPSY gel (GTPSY) were characterized mechanically (Figure 3) for their hardness, adhesiveness, and cohesiveness. The height of the positive peak on the force time curve gives the hardness of the formulation. It indicates the resistance to compression indicating the ease by which product can be removed from the container. The hardness of GTPSY was found to be more than that off GPSY. The adhesiveness of the formulation, that is, area of the first negative peak on the force time curve, measures the work required to overcome the forces of attraction between the probe and gel surface. The results revealed higher adhesiveness of GPSY as compared to GTPSY. The cohesiveness of the formulations is calculated from the ratio of the area under second positive peak to the first positive peak. It indicates the structural recovery of the gel formulation after compression. The cohesiveness of GTPSY was found to be more than that of GPSY. The GTPSY gel shows more hardness and cohesiveness. The area of the negative peak of the force time curve measures the adhesiveness of the formulation. It indicates the work required to overcome the forces of attraction between the gel surface and the probe. 

Table 1 summarizes the results of mucoadhesive strength, *ex vivo* bioadhesion, and swelling studies conducted using metronidazole-loaded PSY and TPSY gel. It is desired that gel formulation intended for buccal delivery should have low hardness but high cohesiveness so that the formulation can be easily removed from the container and it recovers its structure completely after application. Further, for prolonged retention in buccal cavity the formulation should have higher adhesiveness. The gel formulation prepared employing GTPSY had higher hardness and lower adhesiveness but more cohesiveness for the formulation. However, the adhesiveness (force of detachment between probe and gel surface) measured using texture analyser cannot be considered as true indicator of mucoadhesive potential. Thus in order to observe the real mucoadhesive potential of the formulation GTPSY and GPSY were comparatively evaluated for their mucoadhesive potential using fresh chicken buccal mucosal membrane. It was observed from the mucoadhesive study carried out by using modified physical balance method that the force required for the detachment of chicken buccal mucosal membrane from GTPSY was threefold higher than that from GPSY.

The mucoadhesive potential of psyllium and thiolated psyllium was evaluated comparatively by measuring mucoadhesion retention time, employing dye containing psyllium and thiolated psyllium gels. A retention time of 7 h and 10.5 h was observed for psyllium and thiolated psyllium, respectively. Thus a 1.5-fold higher mucoadhesion retention time was observed on thiolation of psyllium.

**Figure 4:**
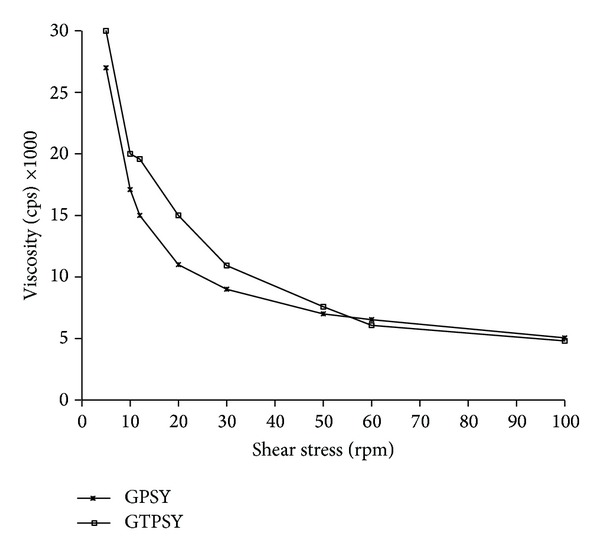
Viscosity of GPSY and GTPSY.

Figure 4 compares the viscosity of gel formulations measured using Brookfield viscometer. The results show no significant difference between the viscosity of PSY and TPSY. 

**Figure 5:**
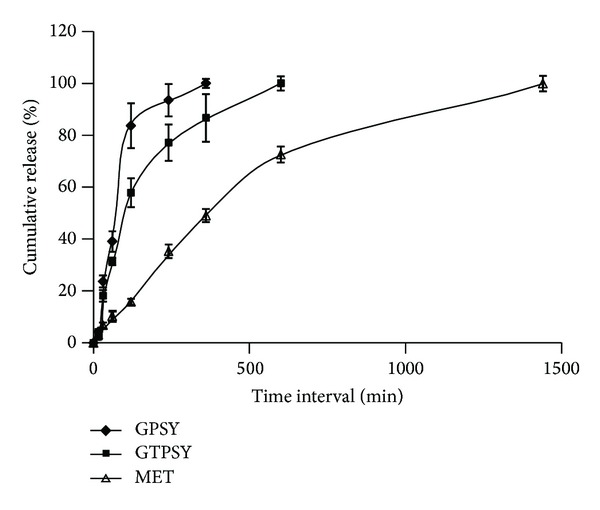
*In vitro* release profile of metronidazole from GPSY, GTPSY, and Metrogyl gel.

Figure 5 displays the *in vitro* release profile of metronidazole from GPSY, GTPSY, and commercial metronidazole gel formulation (Metrogyl). It can be inferred from the plot that GTPSY provided a slower release of metronidazole in comparison to TPSY. Even though there was no significant difference between the viscosity of GPSY and GTPSY, the GTPSY provided a prolonged release of metronidazole. This could be due to the *in situ* cross-linking exhibited by disulfide linkages of thiolated polymer. The commercial formulation Metrogyl provided a prolonged release over a period of 24 h. However, by optimizing the concentration of GTPSY and the degree of thiolation a gel formulation with desired release characteristics can be easily formulated. 

The release of metronidazole loaded gels and marketed formulations was fitted into various kinetic models to estimate their release kinetics and mechanism of release (Table 2). The results of release rate data for all the gel formulations fitted best into first-order release kinetics. Further, the value of “*n*,” the release exponent of Korsmeyer and Peppas equation, indicates that the release of metronidazole from GPSY and GTPSY (*n* > 0.5) occurs by combination of polymer relaxation and diffusion through the polymeric matrix while the release from commercial formulation occurs by diffusion through the matrix [12].

## 4. Conclusions

Thiol modification of psyllium was carried out by esterification with thioglycolic acid. Thiolated psyllium was characterized by FT-IR, DSC, XRD, and SEM study. Modified psyllium was employed for formulating mucoadhesive gels using metronidazole as the model drug. Thiolation of psyllium resulted in 3-fold increase in its mucoadhesive strength and 1.5-fold increase in mucoadhesion retention time. The results of *in vitro* release study indicate that thiolation of psyllium imparts sustained release characteristics without affecting its viscosity. In conclusion, thiol modification of psyllium improves its mucoadhesive properties.

## Figures and Tables

**Table 1:** Evaluation of mucoadhesive strength, swelling power, and mechanical parameters of gels.

Formulation	Mucoadhesive strength (N)	Hardness (N)	Adhesiveness (g sec)	Cohesiveness	Swelling power (mL)
GPSY	0. 1205 ± 0.02	0.093	−36.80	8.8	86.6 ± 0.01
GTPSY	0.3494 ± 0.04	0.1972	−54.89	18.8	100 ± 0.03

**Table 2:** Modeling and release kinetics of metronidazole gel formulations.

Formulation	Zero-order *R* ^2^	First-order *R* ^2^	Higuchi *R* ^2^	Korsmeyer-Peppas	
				*R* ^2^	*n*
GPSY	0.817	0.964	0.925	0.896	0.798
GTPSY	0.827	0.988	0.956	0.921	0.514
Metrogyl	0.896	0.989	0.974	0.739	0.291
